# Cognitive Impairment Beyond Neurodegenerative Dementias: New Frontiers for Cognition in Neurological Disease

**DOI:** 10.1111/ene.70614

**Published:** 2026-04-29

**Authors:** Paulo Caramelli, Isabel Pavão Martins

**Affiliations:** ^1^ Behavioral and Cognitive Neurology Unit, Faculdade de Medicina Universidade Federal de Minas Gerais Belo Horizonte (MG) Brazil; ^2^ Centro de Estudos Egas Moniz, Faculdade de Medicina Universidade de Lisboa; Hospital de Santa Maria, ULS Santa Maria Lisboa Portugal

Cognitive impairment is a frequent finding in neurological disorders and is an important clinical feature to consider for diagnosis and care of neurological patients [1, 2, 3]. Neurodegenerative diseases like Alzheimer's disease, Lewy body diseases (i.e., Parkinson's disease and dementia with Lewy bodies) and frontotemporal dementia, as well as cerebrovascular disorders, have received significant attention in the last decades, due to their high prevalence rates and their impact on patients, families and societies [4]. However, an increasing body of research is now shedding light on cognitive deficits emerging from a diverse range of other causes, some of which remain undervalued or under investigated. This special issue of *The European Journal of Neurology* presents 19 original and review manuscripts that dive into this less explored territory, offering valuable insights into cognitive impairment beyond traditional dementias.

One published study investigates cognitive deficits in patients with peripheral vestibular dysfunction, a common condition in Neurology, Otolaryngology, and Internal Medicine clinics, but usually not present in discussions and in research in the area of Cognitive Neurology. Additionally, the persistent effects of post‐COVID‐19 syndrome on cognition are examined in several studies, addressing topics ranging from the subjective experience of brain fog to systematic reviews and meta‐analyses on the patterns of cognitive deficits after COVID‐19.

The issue also includes manuscripts exploring the cognitive burden experienced by individuals with multiple sclerosis (MS), presenting interesting findings about the discordance between patients' and clinicians' perceptions of cognitive issues, and the effect of cannabis on cognition in persons with MS. There are also three interesting studies reporting data from cognitive tools and the potential for electronic tools to monitor cognitive function in MS, as well as one study investigating a sub‐population of lymphocytes and cognitive dysfunction. In addition, one case‐control study compares cognitive performance of patients with myelin oligodendrocyte glycoprotein antibody‐associated disease to MS patients. Taken together, these studies challenge existing diagnostic paradigms and highlight the need for more accurate and comprehensive cognitive assessments.

Functional cognitive disorder, a condition that is placed at the intersection of Neurology and Psychiatry, is the object of two manuscripts, one international Delphi consensus on diagnosis and management, and a systematic review with meta‐analysis about cognitive performance in people with the disorder. Another study uses electroencephalographic analyses to investigate the effects of sub‐concussion in boxers. Cognitive aspects in subjects on hemodialysis and in individuals with spinocerebellar ataxia type 3 are also displayed.

These manuscripts contribute to expanding the boundaries of cognitive research to encompass a broader array of conditions. This issue underscores the importance of considering diverse etiologies and patient experiences in the diagnosis and management of cognitive impairment. In fact, given the widespread and interconnected organization of cognitive functions across brain regions, it is no surprise that they may potentially be affected by any brain disorder. We believe that these studies may contribute to a more comprehensive understanding of cognitive deficits in neurology, emphasizing that cognitive impairment is not confined to neurodegenerative and cerebrovascular diseases.

## Author Contributions

**Paulo Caramelli:** conceptualization, writing – original draft, writing – review and editing. **Isabel Pavão Martins:** conceptualization, writing – original draft, writing – review and editing.

## Conflicts of Interest

The authors declare no conflicts of interest.

## Data Availability

Data sharing not applicable to this article as no datasets were generated or analysed during the current study.
